# Ruptured Intracranial Dermoid Cyst with Fat Dissemination: A Clinical Case Mimicking an Epidermoid Cyst and Review of the Literature

**DOI:** 10.3390/diagnostics15060712

**Published:** 2025-03-12

**Authors:** Kalvis Verzemnieks, Roberts Tumelkans, Sintija Strautmane, Verners Roberts Kalejs, Egils Valeinis, Julija Dolgopolova, Tatjana Tone, Arturs Balodis

**Affiliations:** 1 Institute of Diagnostic Radiology, Pauls Stradins Clinical University Hospital, LV-1002 Riga, Latvia; kalvisverzemnieks@gmail.com (K.V.);; 2 Department of Radiology, University of Latvia, LV-1586 Riga, Latvia; 3 Faculty of Medicine, Riga Stradins University, LV-1007 Riga, Latvia; roberts.tumelkans@gmail.com; 4 VCA Polyclinic Plavnieki, LV-1021 Riga, Latvia; sintijasstrautmane@gmail.com; 5 Department of Neurology, Riga East Clinical University Hospital, LV-1038 Riga, Latvia; 6 Clinic of Neurosurgery, Pauls Stradins Clinical University Hospital, LV-1002 Riga, Latvia; egils.valeinis@stradini.lv (E.V.); julija.dolgopolova@stradini.lv (J.D.); 7 Institute of Pathology, Pauls Stradins Clinical University Hospital, LV-1002 Riga, Latvia; airisa13@inbox.lv; 8 Department of Radiology, Riga Stradins University, LV-1007 Riga, Latvia

**Keywords:** epidermoid cyst, intracranial dermoid cyst rupture, intraventricular fat dissemination, subarachnoid fat dissemination, blooming artifact

## Abstract

**Background and Clinical Significance:** Intracranial dermoid cysts (IDCs) are rare benign congenital intracranial lesions. In the case of IDC rupture, these lesions may manifest clinically. Cysts may be visualized on non-enhanced computed tomography (NECT) and magnetic resonance imaging (MRI), facilitating discussions between clinicians and radiologists to determine cyst content and potential dissemination in cases of rupture. This case report describes an IDC rupture presenting as fat-containing lesions in the subarachnoid space and ventricular system, resembling a subarachnoid hemorrhage on MRI. **Case Presentation:** A thirty-two-year-old Caucasian male patient was admitted to the hospital due to recurrent headaches and visual impairment that began at the age of thirty-one. MRI revealed a lesion radiologically consistent with a ruptured dermoid or epidermoid cyst in the anterior fossa with a mass effect on the optic nerve intracranial segments, the chiasma opticum, and proximal optic tracts. The patient underwent a successful neurosurgical resection of the lesion, and histopathological analysis confirmed the diagnosis of a dermoid cyst. The postoperative period was uneventful. MRI follow-up revealed residual tissue of the IDC without any volume increase. Multiple punctate fat-containing lesions were noted, similar to previous MRIs. The patient reported no complaints at discharge. Follow-up MRI imaging demonstrated no recurrence or progression of the dermoid cyst at 4 months, 1 year, and 2 years. **Conclusions:** IDC rupture is a rare event that may present clinically and appear as a blooming artifact on MRI, mimicking subarachnoid hemorrhage. Fat-containing lesions in the subarachnoid space and ventricular system can demonstrate findings indicative of an IDC rupture. MRI diffusion-weighted imaging (DWI) and decreased apparent diffusion coefficient (ADC) values may mimic an epidermoid cyst, a phenomenon rarely described in the literature, further complicating the diagnostic process.

## 1. Introduction

Intracranial dermoid cysts (IDCs) are rare, benign, slow-growing congenital lesions originating from ectodermal elements during neural tube closure, typically between the 5th and 6th weeks of fetal development [1]. By definition, intracranial dermoid cysts are derived from ectodermal tissue. Histologically, dermoids, unlike epidermoids, not only contain squamous epithelium but may also contain hair follicles, stratified squamous epithelium, and apocrine, eccrine, and sebaceous glands [2]. On the contrary, teratomas are neoplastic structures originating from aberrant embryonic germ cells, typically composed of tissues derived from multiple germ layers [2]. IDC differential diagnosis includes epidermoid cyst, lipoma, teratoma, craniopharyngioma, etc. Epidermoids are typically located off the midline, while dermoids are generally found in or near the midline.

According to the available literature, IDCs constitute 0.04–0.70% of all intracranial tumors [3,4]. Intracranial dermoid cyst ruptures account for approximately 0.18% of all surgically treated CNS tumors, based on data from a 12-year retrospective review at the University of Utah School of Medicine [5]. IDCs may develop anywhere in the skull, but they tend to be more common in the middle of the facial/skull bones. Precise data on IDCs in the anterior cranial fossa are lacking. Associated complications of IDC rupture include hydrocephalus, chemical meningitis, etc. In published cases so far, the incidence of IDC complications varies, but the exact data have not been fully elucidated. Further research on this topic is warranted to assess precise data about IDC rupture complications. Ruptures are often described in adults, mostly at around 30–60 years of age, and rarely in children, mostly under 10 years old. Men are slightly more affected than women [3].

On a non-enhanced computed tomography (NECT), IDCs are typically noted as hypodense lesions with fat-density material [1]. In brain magnetic resonance imaging (MRI) studies, dermoids are typically hyperintense lesions seen on T1-weighted images and hyper- to hypointense structures on T2-weighted images [1]. Published studies so far show that IDCs can develop in various locations, and the most common sites include midline sellar and suprasellar, parasellar, posterior fossa, and frontonasal regions. If the size of the cyst affects the surrounding tissues, it may cause various neurological symptoms, such as headaches, seizures, numbness, weakness, visual impairment, neck stiffness, and facial pain. On the contrary, the rupture of IDCs is a rare complication that may lead to severe outcomes, such as aseptic meningitis caused by the leakage of cyst contents into the subarachnoid space. Intracranial dermoid cysts are usually well-defined, lobulated midline masses.

## 2. Case Presentation

### 2.1. Clinical Features

A thirty-two-year-old Caucasian male presented to a tertiary university hospital due to recurrent headaches and paroxysmal visual impairment during the last twelve months. The MRI brain scan revealed a likely benign intracranial dermoid or epidermoid cyst affecting the optic nerve. Initially, the MRI results were unclear and did not distinguish between the two. The patient was consulted by a neurosurgeon, and a planned neurosurgical treatment was scheduled. The patient had no prior documented medical conditions.

A month later, the patient was hospitalized for planned surgery. During this time, the patient claimed his headache and vision disturbances had gotten worse. Due to the patient’s complaints about a decline in visual acuity, Optical Coherence Tomography (OCT) was recommended and performed, which led to the diagnosis of Astigmatismus myopicus in both eyes or oculus uterque (OU). The palpebral fissures of both eyes were symmetrical; eye movements were symmetrical, free, and within normal amplitude in all gaze directions; and no nystagmus was observed. The oculus uterque (OU) fundus oculi—temporal part slightly paler, peripheral atrophy, macula—were within normal limits. The vessels were slightly tortuous. No additional pathological findings were noted. The patient’s overall health condition was stable. Neurologically, the Glasgow Coma Scale (GCS) was 15, and the patient was alert. The pupils were symmetrical and reactive to light. No paresis was noted. The deep tendon reflexes were symmetrical, and no pathological reflexes were seen. The patient did not demonstrate any coordination difficulties. There was no sensory impairment. The patient’s gait was stable. Meningeal signs were negative.

### 2.2. Imaging Findings

**Figure 1:**
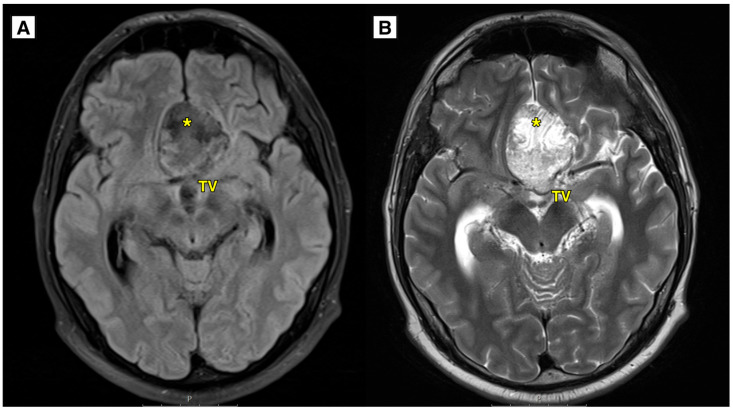
(**A**): MRI of the brain (T2-FLAIR axial sequence) showing a slightly hyperintense lesion, most likely a dermoid cyst, located in the basal dorsal anterior fossa. The cyst extended into the suprasellar cisterns, positioned above the chiasma opticum and the prechiasmal part of the optic nerve. It was situated under the anterior cerebral arteries and caused compression and displacement of the anterior parts of the third ventricle. Despite this, the foramen of Monro remained open. Additionally, there was an expansion of the lateral and third ventricles. (**B**): MRI of the brain (T2W-TSE axial sequence) showing an intracranial cyst located in the anterior fossa. The lateral ventricles were dilated, with the temporal horns measuring up to 8.5 mm, indicative of hydrocephalus. The likely cause of hydrocephalus development was a combination of the ruptured dermoid cyst and its intraventricular spread, exerting a mass effect on the third ventricle and aqueductus cerebri. TV—third ventricle, *—intracranial dermoid cyst.

**Figure 2:**
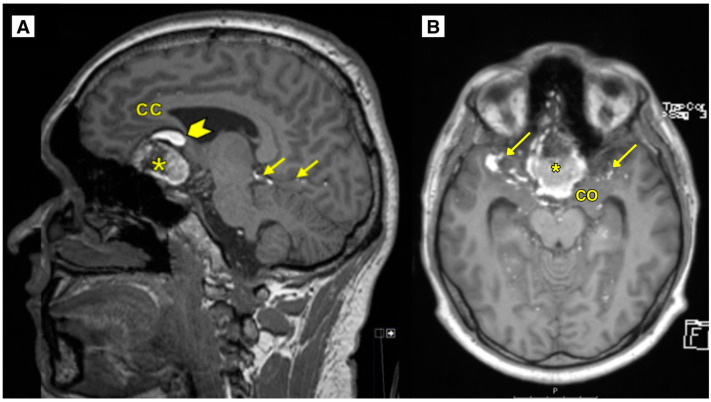
(**A**): MRI of the brain T1W sag. The intracranial dermoid cyst located in the anterior fossa with hyperintense fat-containing lesions (small arrows) and extracellular methemoglobin-containing hemorrhage (arrowhead), representing a small subacute hemorrhage due to cyst rupture. (**B**): MRI of the brain T1W ax. sequence showing the intracranial dermoid cyst located in the anterior fossa with a mass effect on the chiasma opticum. Dermoid cyst rupture with massive subarachnoid fat inclusions of various sizes in the frontal lobes, cisterns, occipital, temporal lobes, and upper parts of the cerebellar hemispheres. CC—corpus callosum, CO—chiasma opticum, *—intracranial dermoid cyst, arrows—fat-dissemination lesions, arrowhead—extracellular methemoglobin-containing subacute hemorrhage.

**Figure 3:**
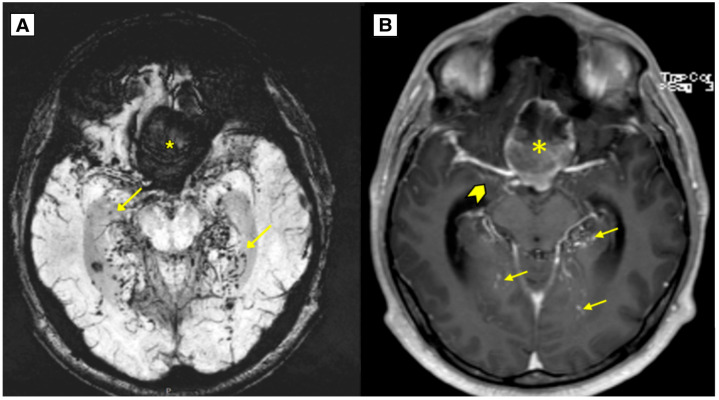
(**A**): MRI of the brain SWI susceptibility-weighted imaging sequence showing the intracranial dermoid cyst located in the anterior fossa with fat-containing lesions, revealing a blooming artifact (yellow arrows). (**B**): MRI T1W post-contrast axial sequence showing leptomeningeal enhancement around the uncus most likely due to chemical meningitis (arrowhead). Arrows—punctate fat-containing lesions, *—intracranial dermoid cyst.

**Figure 4:**
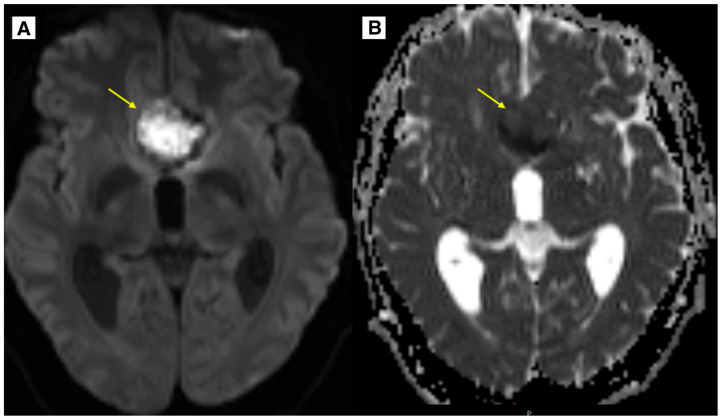
In the images (**A**) (DWI sequence) and (**B**) (ADC map), there is a lesion located in the anterior cranial fossa along the midline, demonstrating slight diffusion restriction and minimally decreased ADC values (yellow arrow). Preoperatively, this lesion mimicked an epidermoid cyst; however, histology confirmed it to be a dermoid cyst.

An MRI brain scan was performed on an outpatient basis. MRI revealed most likely a ruptured dermoid cyst located in the anterior fossa, with a mass effect on the optic chiasm, prechiasmatic part (Figure 1 and Figure 2). The dermoid cyst dimensions were 4.6 × 4.0 × 2.5 cm (AP × LL × CC) (Figure 1). The susceptibility-weighted imaging (SWI) sequence showed hypointensity with a blooming artifact (Figure 3). The radiologist initially considered a diagnosis of a dermoid or epidermoid cyst based on what was seen in the diffusion-weighted imaging (DWI) sequence and apparent diffusion coefficient (ADC) maps, which was later ruled out following the histology results (Figure 4).

### 2.3. Management

**Figure 5:**
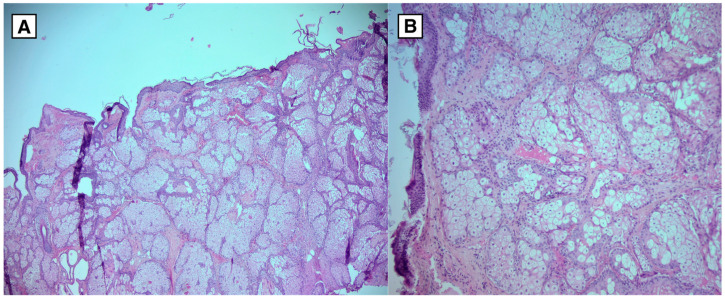
Hematoxylin and Eosin stain of the dermoid cyst. Tissue samples lined by squamous epithelium with keratinization. Subepithelially abundant sebaceous gland foci and hair follicles. The tissue lacks cytological atypia. (**A**): Microscopic magnification 4× (HPF). (**B**): Microscopic magnification 10× (HPF).

The patient underwent a subtotal cyst surgical resection via right-side osteoplastic craniotomy, and histopathological analysis confirmed the diagnosis. The postoperative period was uneventful. Histological examination showed a cyst containing fibrous tissue, multiple squamous epithelium with hyperkeratosis, subepithelial sebaceous glands, hair follicles, focal hemorrhage, and focal lymphocytic infiltration. The morphological appearance corresponded to a dermoid cyst (Figure 5).

**Figure 6:**
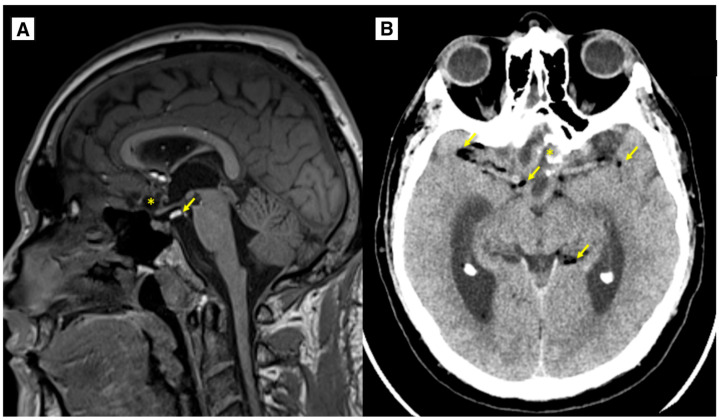
Follow-up. (**A**): MRI of the brain T1W sagittal at the 1-year follow-up, showing that the dermoid cyst has been subtotally resected (*). There are very small fat lesions (yellow arrows), as well as residual calcifications of the dermoid along the left side of the postoperative lobe, and, as in the previous MR examination, small scarring changes with encephalomalacia in the dorsal parts of the gyrus recti of both frontal lobes. No data are provided on the progression of the dermoid cyst. (**B**): CT of the brain at the 2-year follow-up, showing that the intracranial dermoid cyst has been largely resected (*). There are very small fat lesions (yellow arrows). No data are provided on the progression of the dermoid cyst. *—intracranial dermoid cyst, arrows—punctate fat-containing lesions.

The patient was discharged from the hospital without any complaints. The patient’s overall health status was satisfactory; neurologically, the GCS was 15 points, no focal neurological deficit was seen, the patient’s vision improved, and no complaints regarding visual disturbances were reported. The surgical wound healed primarily. No recurrence or dermoid progression was noted on follow-up MRI imaging at 4 months, 1 year, and 2 years. MRI follow-up, performed 1 year after surgery, showed residual tissue of the tumor without volume increase; residual calcifications were also seen along the suprasellar region on the left side. Many punctate fat-containing lesions, both along the ventricle wall and subarachnoid, were similar to the previous MR examination (Figure 6). Scarring changes were observed, with encephalomalacia more in the right frontal lobe in the basal medial parts, extending more at the level of the gyrus recti, with minimal blood products. The optic nerve was not scarred, and no abnormal tissue was found around the optic nerve following neurosurgical treatment. It is important to monitor for any signs of optic nerve atrophy during follow-up MRI (Figure 6).

During follow-up four months after surgery, the patient reported a loss of smell and altered taste. The reason for the loss of smell could be explained by the fact that the dermoid cyst was located in the anterior fossa, and the patient underwent a neurosurgical treatment in this region. Any neurosurgical approaches to the anterior cranial fossa put the olfactory pathway and its connections to other brain structures at risk. On the other hand, the altered sense of taste in this case could be explained by postoperative scarring and encephalomalacia in the right frontal area and in the dorsal parts of the gyrus recti of both frontal lobes.

Craniotomy can provoke seizures in a small proportion of patients, and the literature data about the incidence are inconsistent. In this case, the patient did not report having seizures before the neurosurgical treatment.

During a follow-up four months after the neurosurgical treatment, the patient had two seizure episodes, most likely provoked by ethanol intoxication. Seizures related to alcohol are a common finding. To rule out possible epileptic activity, an EEG was performed, demonstrating focal changes in the right frontal area, possibly indicating an epileptiform focus. However, a follow-up MRI DWI showed no indications of possible ictal injury. The early postoperative period in this case was uneventful, and the average recovery time after the craniotomy was up to two months. While seizures in this patient were observed only at a follow-up four months after the neurosurgical treatment, provoked by ethanol intoxication, it is unlikely that there was an epileptic activity in the early postoperative period.

The observed signal characteristics could be explained by the lesion being keratin-rich, which can cause diffusion restriction. Additionally, it is hypothesized that the fatty content of the dermoid cyst was less dense, contributing to the atypical imaging appearance.

## 3. Discussion

Intracranial dermoid cysts, also called dermoid tumors, are congenital benign cystic formations. The incidence of intracranial dermoid cysts is reported to be 0.04–0.70% of all intracranial tumors [3,4].

Intracranial dermoid cyst ruptures account for approximately 0.18% of all surgically treated CNS tumors, based on data from a 12-year study in a major clinic [4]. Ruptures are most often described in adults (commonly 30–60 years) and rarely in children (commonly under 10 years). Men are slightly more affected than women [3]. The age and gender of the patient align with these epidemiological trends.

Ruptures may occur spontaneously or during intracranial surgery, or they may present after trauma [6]. Ruptures of brain dermoid cysts are uncommon; therefore, little is known about their pathogenetic mechanisms. Some authors suggest rapid cyst growth due to age-related hormonal changes or the rapid expansion of small cysts against the resistance of their capsule [7,8]. Others dispute this theory, noting that the majority of ruptures happen between the ages of 30 and 50, when hormonal changes are no longer significant [7,8].

More than 30 similar publications on intracranial dermoid cysts have been published so far. Out of those, cases of dermoid cyst ruptures are described in selected publications. Illustrative materials and detailed radiological findings are offered even more seldom. In 16 of these publications, cases of anterior cranial fossa dermoid cyst ruptures with fat dissemination into the subarachnoid space have been described specifically. In these reports, the patient group enrolled 16 people, where 10 of them were men, and the age ranged between 21 and 60 years, with the highest incidence of these cyst ruptures in the 30–50-year-olds. According to the statistics, supratentorially located dermoid cysts are the most frequently reported, with anterior cranial fossa cysts accounting for 29.6% of the cases [5]. They are often located parasagittally on the basal portion of the anterior cranial fossa [9].

### 3.1. Clinical Features

The clinical picture is variable due to the location of the cyst rupture. The most commonly noted symptoms include sudden, excruciating, or prolonged progressive headaches, mostly in younger patients (31.8%) [9,10,11,12,13,14,15,16], generalized or focal seizures in older individuals (29.5%) [3,14,17,18], impaired consciousness or awareness [11,19,20], and focal symptoms of CNS lesions (16.3%) [14]. In this case, the main complaints of the patient included a prolonged headache and vision impairment, which were followed by gradual improvement.

When fat tissue enters the subarachnoid and ventricular systems, it results in obstruction and hydrocephalus. Some authors also highlight the mass effect of the cyst as a compressive factor [21]. Liu J.K. et al. observed the development of hydrocephalus in 29% [5] of all cases of brain dermoid cyst ruptures. Case reports indicate the formation of hydrocephalus both in the early days of the illness and in the late postoperative period [19,21,22].

The fat-containing cyst materials directly irritate cerebral blood vessels, resulting in vasospasms, which can act as a trigger for transient brain ischemia or cerebral infarction [4,15].

### 3.2. Imaging Findings

All literature sources indicate computed tomography (CT) and magnetic resonance imaging (MRI) as diagnostic methods of choice in cases of dermoid cyst ruptures in the anterior cranial fossa. Imaging characteristics relate to specific signal intensities of the cyst contents, lack of edema around the lesion site, and clear visibility of defined cyst borders [23]. The best diagnostic indicators suggesting dermoid cyst rupture are the dissemination of fat droplets in the subarachnoid space, brain sulci, and ventricles [4].

Brain CT scans show low-density lesions corresponding to the fatty content of the cyst fluid. Hypodense foci in the subarachnoid space and ventricular system indicate the dissemination of fat droplets after cyst rupture [4,21]. In CT examinations, the density of fat corresponds to negative Hounsfield units. However, the density value may be higher than typical for fat due to the influence of a dermoid cyst’s contents [15]. Clinical cases describe Hounsfield unit values of 87 and 97 at the site of the lesion [9,10]. When the lipid content dominates at the lesion site, CT scans show homogeneous hypodense changes, while heterogeneity is determined by cyst inclusions, such as hair derivatives, calcifications, and epidermal remnants [21].

According to the literature, peripheral calcification of the cyst capsule is detected in 20% of intracranial dermoid cysts during CT scans [24]. The mass effect of the cyst is typically minor compared to the size itself [23]. In the clinical cases described in the available literature sources, contrast was not used during CT examinations.

In magnetic resonance imaging (MRI) examinations of intracranial dermoid cyst ruptures, a characteristic hyperintense lesion is observed on T1-weighted images due to fat content, while a heterogeneous lesion appears on T2-weighted images, which is related to various cyst inclusions (e.g., hair, calcifications, nails, and epidermal remnants) [25,26,27]. A high-intensity signal in the subarachnoid space and ventricular system on MRI indicates fat dissemination following dermoid cyst rupture [21]. T1W1 is the best sequence for detecting fat droplets in the subarachnoid space in cases of cyst rupture. In FLAIR sequences, a ruptured dermoid cyst also appears hyperintense, while GRE sequences reveal a blooming artifact in the cyst mass and surrounding area [10]. The examination is performed with intravenous gadolinium contrast. A signal intensity similar to cerebrospinal fluid can be seen if the inner fat content of the cyst is fairly low [15].

The blooming artifact is commonly observed at the periphery of fat droplets as a dark, narrow rim around the fat globule [28]. This phenomenon arises from the interaction of fat and water with the resonance frequency signal, leading to its spreading beyond the boundaries of the structure. The hydrogen protons in fat and water create a resonance frequency difference, which can result in signal intensity loss in these proton pixels due to mutual cancellation. This effect produces the characteristic appearance of a dark rim around the fat globule and, in phase images, may generate significant phase shift contrasts. The role of the susceptibility gradient at the fat-tissue interface in this process is also noted [29,30].

In this case, typical radiological features were observed, as well as a blooming artifact indicating a fat-containing lesion in the subarachnoid space and ventricular system (Figure 3). These low-signal foci in the T2 and SWI sequences must be differentiated from acute subarachnoid hemorrhage or hemosiderin inclusions. To aid in detecting blood products in the brain, the SWI sequence is used, as it is much more sensitive to hemosiderin inclusions, resulting in a more weighted T2W series [29,31]. Residual calcifications of the dermoid may be seen along the front and left side of the postoperative lobe on a follow-up MRI one year post-surgery (Figure 6).

The results of the MRI sequences revealed not only typical radiological features of dermoid cysts but also findings that need to be differentiated from other diseases. The hyperintensity on DWI with reduced diffusion mimicked an epidermoid cyst (Figure 4); T2-FLAIR and T1W sequences showed blood deposits in the brain (Figure 1 and Figure 2).

In the available English-language literature, there are only a few clinical case reports describing findings typical of epidermoid cysts in brain MRI DWI sequences that have been identified in cases of dermoid cysts. Johnson D.G. et al. present a clinical case where a DWI examination revealed reduced diffusion [20]. A high-intensity DWI signal with reduced diffusion was also reported in the study by Aksoy F.G. et al. [32]. Furthermore, the literature includes a reference to a similar finding described by Japanese researchers Shinoyama M. et al. [33]. In this clinical case, the DWI sequence and ADC map demonstrated slight diffusion restriction and a minimally decreased ADC value. Such radiological findings are characteristic of epidermoid cysts. The epithelial cells of cyst walls secrete the protein keratin, which also contributes to the reduction in the diffusion observed on DWI. Dermoid cysts also contain keratin, and if the fat content within the cyst is low, it can mimic the radiologic features characteristic of epidermoid cysts. This can potentially lead to diagnostic errors in the preoperative period [32,34]. Since these findings are rarely discussed, they represent some of the novel contributions of this article.

The keratin-rich nature of the lesion, which can result in diffusion restriction, may account for the observed signal characteristics. Furthermore, the unusual imaging appearance is thought to have been caused by the dermoid cyst’s less dense fatty content (Figure 4).

A histological examination of surgical material confirms the diagnosis, particularly in cases with atypical radiological findings. Histological samples of intracranial dermoid cysts reveal keratinized squamous epithelium containing sebaceous glands, sweat glands, hair follicles, and other ectodermal structures. In contrast, epidermoid cysts have a stratified squamous epithelium with no skin derivatives.

### 3.3. Management

Summarizing the available information about the treatment of a ruptured dermoid cyst in the anterior cranial fossa, surgical cyst resection is the main choice, as well as the use of intravenous glucocorticoids, which pathogenetically affect inflammation mediators. Removing the capsule and cyst contents is the aim of the procedure [19]. Complete evacuation of fat droplets from the subarachnoid space and ventricular system, considering their diffuse dissemination across both hemispheres, is usually not possible [19]. Authors also highlight limitations for total resection of dermoid cysts: proximity of the cyst capsule to surrounding neurovascular structures, which prevents separation of the cyst capsule without causing neurological damage [19]. In the reviewed clinical cases, only one involved a subtotal resection of a dermoid cyst [11]. A particularly technically challenging operation is described in the resection of a ruptured dermoid cyst due to the rupture of a cerebral artery aneurysm, where the cyst capsule was separated from the aneurysm’s edge [11].

Considering the close association of the dermoid cyst with the optic nerve and the anterior cerebral artery, a subtotal cyst resection was performed in this clinical case. Authors describe recurrences of dermoid cysts after subtotal resection as very rare [19,20,21], which is also confirmed by this clinical case—over a two–year period, no recurrence of the dermoid cyst was observed.

In this case, the patient underwent a subtotal resection of the cyst. In the literature, such a procedure is performed extremely rarely [19,20,21]. This case report not only provides highly illustrative material of intracranial dermoid cyst rupture but also raises the knowledge of intracranial dermoid cyst rupture, adding to the very limited number of case reports available.

In cases of chemical meningitis, high-dose glucocorticoids administered intravenously in a hospital setting have been described for treatment and prevention, with continuation of oral administration on an outpatient basis [21]. High doses of intravenous dexamethasone are already initiated in the preoperative period [21]. The conservative treatment of intracranial dermoid cyst ruptures is also based on the use of high-dose intravenous glucocorticoids in the acute period, with a gradual tapering over several months [10].

## 4. Conclusions

Intracranial dermoid cyst rupture is a rare occurrence that might manifest clinically and show up as a blooming artifact on MRI, resembling subarachnoid hemorrhage. Fat-containing lesions in the subarachnoid space and ventricular system can demonstrate findings suggestive of an IDC rupture. Additionally, diffusion restriction on DWI and decreased ADC values may mimic an epidermoid cyst. This phenomenon has rarely been described in the literature, further complicating the diagnostic process. Therefore, it is important to perform examinations using multiple sequences for a more accurate diagnosis.

## Data Availability

The original contributions presented in this study are included in this article; further inquiries can be directed to the corresponding author.
